# Patients’ and Providers’ Perspective of a Multi-level Approach to Improve Participation in Low-dose CT for Lung Cancer Screening (Empower LCS): A Mixed-Methods Analysis^☆^

**DOI:** 10.1016/j.acra.2026.03.014

**Published:** 2026-03-28

**Authors:** Maedeh Sharifian, Michael A. Hoyt, Aarushi Madan, Sunmin Lee, Tan Q. Nguyen, Gelareh Sadigh

**Affiliations:** Department of Radiological Sciences, University of California, Irvine, CA (M.S., A.M., G.S.); Department of Population Health and Disease Prevention, University of California Irvine, Irvine, CA (M.A.H.); Department of Medicine, University of California Irvine, Irvine, CA (S.L.); Department of Family Medicine, University of California Irvine, Irvine, CA (T.Q.N.).

**Keywords:** Lung cancer screening, Multi-level intervention, Qualitative interviews, Patients, Providers

## Abstract

**Rationale and Objectives:**

Lung cancer screening (LCS) utilization remains suboptimal. We examined the experience of patients and primary care providers (PCPs) participating in a multilevel intervention addressing LCS barriers.

**Methods:**

Empower LCS is a single-arm pilot feasibility trial evaluating feasibility, acceptability and potential impact of a multilevel intervention, including a decision aid, patient reminders, PCP electronic medical record (EMR) notifications, and support for health-related social needs (HRSNs) for patients screening positive for HRSNs, to improve LCS completion. Using a mixed-methods design, quantitative surveys assessed the helpfulness of intervention components. Additionally, 11 patients and five PCPs participated in 30-minute semi-structured interviews, analyzed through a theoretically driven thematic approach.

**Results:**

Of 70 participants, 41 completed experience questions at 6-months (mean age 61.7 ± 6.4; 63.4% male). Most patients found the intervention helpful: 61.5% (24/39) decision aid, 67.5% (27/40) patient reminder, and 70.7% (29/41) PCP’s EMR notification. Most patients viewed LCS as important and wanted to discuss it with their provider. However, some patients felt providers were hesitant to engage fully or lacked knowledge about LCS eligibility. Patient-reported barriers included scheduling challenges, insurance delays, and smoking-related stigma. Many said Empower LCS increased awareness and motivation. Providers reported routinely discussing smoking history and identified low patient awareness, insurance issues, competing priorities, and patient radiation concerns as barriers. They found the intervention appropriate for primary care, especially with better patient education and EMR notifications.

**Conclusion:**

Both patients and providers were receptive to LCS and thought the Empower LCS intervention had the potential to increase LCS in routine practice.

**Trial registration:**

This study is registered at www.clinicaltrials.gov: NCT06000683

## INTRODUCTION

Lung cancer is the leading cause of cancer mortality in the United States. Lung cancer screening (LCS) with low-dose computed tomography (LDCT) decreases lung cancer mortality by 20% compared with chest radio-graphs (1). The 2021 United States Preventive Services Task Force (USPSTF) recommends annual LDCT for adults aged 50–80 years with ≥ 20-pack-year smoking who currently smoke or quit within past 15 years (2). LCS is covered by most insurers without cost-sharing.

Despite benefits and coverage, LCS remains underutilized; only 18.2% of eligible individuals received LCS in 2022 (3). Provider barriers include limited guideline familiarity (4–8), incomplete electronic medical record (EMR) smoking history (8), time constraints (9–11), and concerns about false positives (8). Patient barriers include insurance coverage uncertainty (12), cost concerns (12,13), lack of symptoms, fear of cancer diagnosis, smoking stigma, and access barriers (13).

We developed Empower LCS, a multilevel intervention designed for routine primary care workflows, comprising: (1) a patient-facing decision aid, (2) automated patient text reminders prompting LCS discussion with primary care providers (PCPs), (3) EMR-based PCP alerts of patient eligibility, and (4) screening for and addressing health-related social needs (HRSNs). We evaluated the feasibility, acceptability and potential impact of Empower LCS on LCS completion in a single-arm pilot trial (clinicaltrials.gov: NCT06000683) (14).

This study examines patients’ and PCPs’ experiences with Empower LCS using a mixed-methods design and identifies opportunities for optimizing implementation in real-world practice.

## METHODS

The study was approved by the Institutional Review Board and conducted in accordance with the Declaration of Helsinki. Participants provided informed consent and Health Insurance Portability and Accountability Act (HIPAA) authorization.

### Study Population

The Empower LCS trial enrolled 70 English-, Spanish-, and Vietnamese-speaking patients aged 50–80 years meeting the 2021 USPSTF LCS eligibility criteria. Patients were recruited from academic, community, and federally qualified health center (FQHC) practices of a Southern California health system (14).

As previously described (14), patients were approached via email, mail, telephone, or in-person between November 2023 and September 2024. Consenting participants received the multilevel Empower LCS intervention, completed a six-month follow-up survey, after which they received a $15 gift card.

**Figure 1.**
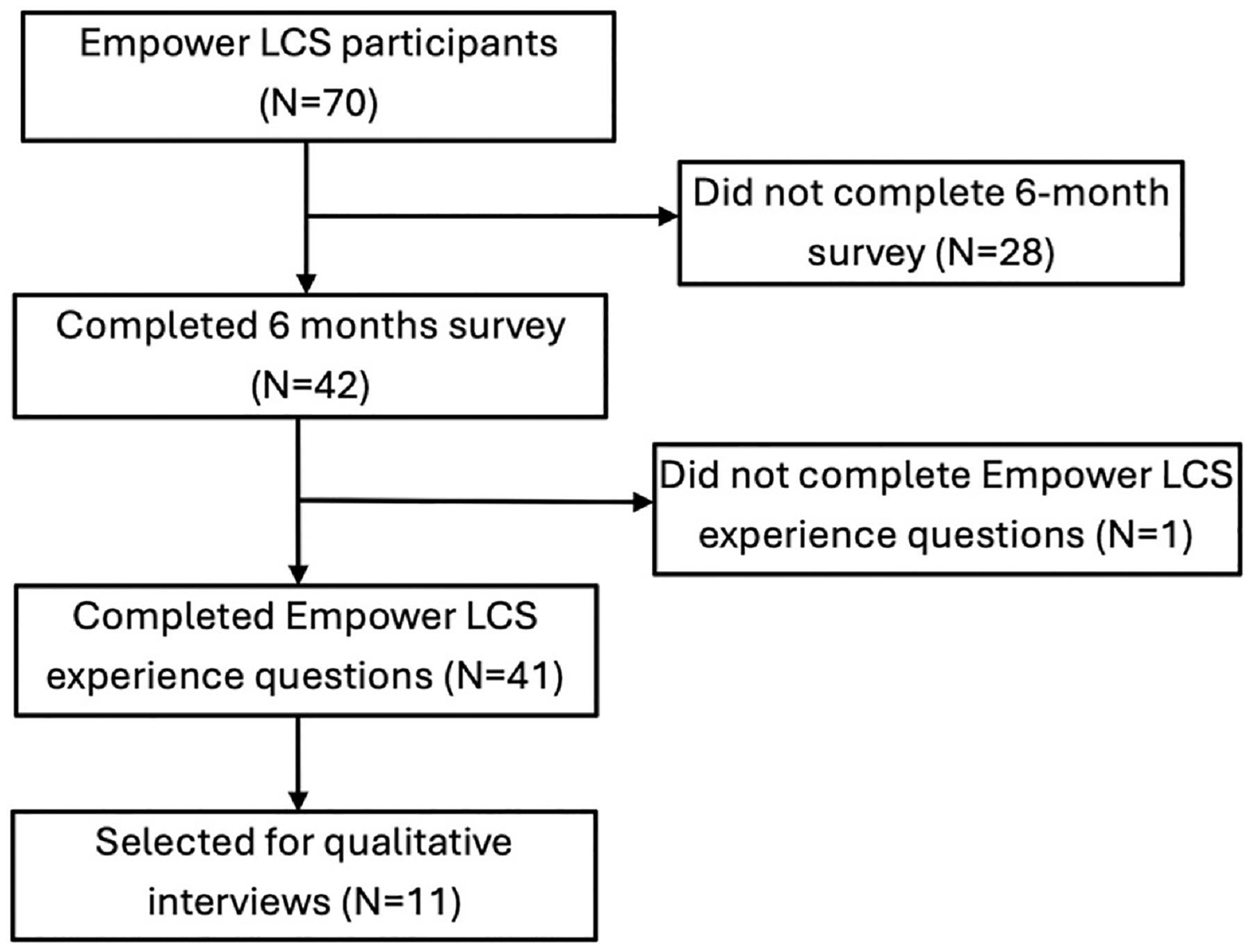
Study Patient Flow Chart.

For this mixed-methods analysis, patients who completed the follow-up survey items evaluating their experience with Empower LCS were included in the quantitative sample (n=41) (Fig 1).

Patients were invited to participate in qualitative interviews within three months of survey completion. We used purposive sampling to reflect linguistic diversity in the study population, selecting five English-speaking, five Spanish-speaking, and one Vietnamese-speaking participants for interviews (n=11).

PCPs were recruited through email. We interviewed five PCPs with patients enrolled in the Empower LCS trial.

### Quantitative Outcomes

Six-month survey assessed patients’ perceived helpfulness of each Empower LCS intervention component using a five-point Likert scale. Responses of extremely, very, or somewhat helpful indicated helpfulness.

### Qualitative Interviews

One-on-one semi-structured interviews (July–November 2024) explored experiences with intervention, barriers and facilitators to completing (patients) or ordering (providers) LDCT, and suggestions for improvement. Interview guides were informed by the Reach, Effectiveness, Adoption, Implementation and Maintenance (RE-AIM) framework (15), focused on implementation-relevant outcomes (Supplemental Materials 1 and 2). Interviews (~30 min) were conducted via videoconference in patients’ preferred language by bilingual trained research staff, with prior qualitative interviewing experience who completed study-specific training focused on use of the interview guide, ethical conduct, and fidelity to the interview protocol. There was an ongoing supervision by a senior researcher with extensive research and clinical interviewing expertise. Thematic saturation was considered achieved when successive interviews yielded no substantively new themes and primary thematic conclusions became redundant. All interview guides were translated by a certified translator. Spanish and Vietnamese interview transcriptions were translated back to English by a certified translator for analysis. Participants received a $40 gift card.

### Data Analysis

Descriptive statistics summarized baseline characteristics and patient experience outcomes. Audio-recorded interviews were translated (as needed), transcribed verbatim, and analyzed using a framework organizing approach. An initial codebook was developed based on the study aims and inductive codes generated from open coding of transcripts. The coding framework was refined through iterative team discussion to ensure conceptual clarity and consistency (16). Coded data were organized into node reports to support identification of patterns and subthemes. Participant-level data were further summarized using matrix displays, incorporating illustrative quotations to highlight key findings (17).

## RESULTS

### Study Population

Of 70 Empower LCS participants, 42 completed the follow-up survey, and 41 completed items assessing their experience with the intervention (Figure 1). Mean age was 61.7 ± 6.4 years, and 63.4% (26/41) were male. Eleven patients were selected for qualitative interviews (mean age: 59; 73% male). Baseline characteristics are shown in Table 1.

### Quantitative Outcomes

At six-month follow-up, most participants rated the intervention as helpful: 61.5% (24/39) for the decision aid, 67.5% (27/40) for the patient text reminder, and 70.7% (29/41) for the PCP notification about screening eligibility.

Overall, 73.2% (30/41) screened positive for financial hardship or HRSNs. Among these, 50.0% (15/30) found the self-referral financial navigation resource helpful.

### Qualitative Patient experience

Major parent codes from patient interviews are summarized in Table 2, reflecting five pragmatic coding categories. Cross-code analysis identified four overarching themes describing patient experience with Empower LCS and engagement with LCS.

#### Theme 1: Raise the Importance, Raise the Motivation

Patients described a proactive stance toward LCS after trial participation. Personal risk factors including smoking history, family history of lung cancer, and occupational exposures were commonly cited as drivers of interest in LCS, with many emphasizing early detection. While most participants recognized the importance of screening, the degree of engagement varied. Some had already discussed LCS with their providers or completed the scan, whereas others had not yet acted on their intentions and anticipated their provider would initiate the conversation. As participant 4646 describes:

“Being invited to participate in this study was actually motivating to me to ask my provider about it [LCS] and for getting the screening done…. So, it was kind of motivational for me to be asked to participate in this study.”

(#4646)

To strengthen LCS motivation and uptake, participants suggested improving provider communication, reducing logistical barriers, and expanding outreach. Reactions to text reminders varied. Some found them helpful, particularly in underserved communities, while others who were proactive in scheduling screenings perceived them as less impactful.

#### Theme 2: Your Screening is Not Like My Screening

Participants described variations in screening experiences. For some, screening was straightforward and efficient, reinforcing a sense of confidence in both the process and their decision to participate. Others encountered barriers including scheduling delays, confusion about where to complete the scan, screening in non-traditional environments such as medical tailors, or difficulty reaching specialists after a positive test. These experiences created uncertainty and shaped expectations about follow-up care. As described:

“Getting the appointment scheduled was pretty easy. I guess when I went and found out that it was going to happen in a trailer outside the building, I think that was a little unexpected.”

(#4646)

Emotional reactions layered onto these logistics. Several participants described anxiety linked to their smoking history or lung cancer risk, which persisted during screening encounter. Positive interactions with imaging technologist or smooth procedure helped relieve this anxiety.

“The feelings and thoughts were internal for me, as you know, for someone who smoked as long as I did and had stopped many years ago there’s always that feeling of, well, ‘what if?’. I took that to the experience itself, with the tech, but it was fine. It was perfect.”

(#3910)

#### Participant Theme 3: Doctor Should Know Best

Participants repeatedly emphasized the central role of PCPs in facilitating LCS. Patients described LCS as a decision that should be guided by a knowledgeable provider who can proactively determine eligibility, initiate conversations, and help navigate insurance and administrative requirements. Although many reported positive experiences characterized by effective communication and shared decision-making, others expressed uncertainty when providers did not clearly explain the next steps or interpret results. Several participants described relying on personal initiative to understand their screening outcomes. For example, one participant reviewed their own results through the patient portal and turned to online sources due to limited explanation:

“When I did talk to the provider, um, I am fortunate because I can go into MyChart and kind of see results there ahead of time, but didn’t see anything concerning in the radiologist report or whatever. They just basically said ‘come back in a year’. So, there were a couple of spots and a couple of slices in a couple of places, uh, the doctor didn’t know, so I’m just going looking at Doctor Google online.”

(#4646)

Participants preferred providers who take time to outline screening steps, discuss the significance of findings, and follow up on care plans. Many felt a proactive approach from the provider could reduce confusion and increase confidence in the screening process. As one participant reflected:

“It seems like the doc could inform patients… it seems like doctors are not reaching out when people are eligible. Seems to me to be a good idea that doctors tell their patient they might be eligible.”

(#3037)

Participants also supported the use of PCP reminders in Empower LCS, interpreting them as a useful prompt to encourage provider-led discussions rather than shifting responsibility to the patient.

#### Theme 4: There are Mountains In Our Way

Despite strong interest in LCS, participants described multiple barriers, including health system processes, insurance and financial constraints, and social and culture dynamics related to smoking history and language.

System-level barriers were particularly common. Participants described difficulty scheduling timely appointments, navigating referral requirements, and experiencing delays due to insurance authorization or clinic workflows. Several participants described frustration after initiating screening discussions with their provider but not being able to follow through due to administrative hurdles beyond their control.

“It’s a pain in the ass to get a doctor’s appointment. They always want to give you one, like, three months out. Which, if you’re sick now, why have you got it three months out? I just don’t get it. And they don’t really spend time with you.”

(#2658)

Insurance-related barriers also emerged, especially for patients with public insurance. Participants reported uncertainty about coverage, facing additional steps to obtain referrals, or being limited in imaging locations due to insurance network restrictions. These barriers were described as discouraging and more burdensome for patients with lower income.

“Getting screening is not easy because you have to have a referral and then they want to see you to give that to you. And it’s not necessarily always easy. And it also depends on insurance. I’m low income, I have Medi-Cal, Medicare -so some things just aren’t available for people that are not sickening rich.”

(#2658)

Participants also described psychological and cultural barriers, including stigma related to smoking and discomfort discussing risk factors. Language barriers were notable among Vietnamese-speaking participants, who struggled with medical terminology, and often relied on family for interpretation.

### Qualitative Provider Experiences

Five PCPs participated in the qualitative interviews: three family medicine faculty, one family medicine resident, and one nurse practitioner. Four practiced in community or FQHC clinics, and one in an academic clinic. Years of practice ranged from 1 to 28 years. Major parent codes from provider interviews are reported in Table 3 reflecting four pragmatic coding categories. Analysis of inter-related areas and thematic coding revealed three key thematic conclusions.

#### Provider Theme 1: Patients are Willing, But Routine Practices Vary

Providers generally described patients as receptive to LCS, particularly those with high trust in the system. Many reported routinely discussing smoking history, especially with older patients, as the starting point for a conversation about LCS.

“*Very few of my patients will bring it forward to me. I can’t think of ever having a patient saying, I need lung cancer screening. I’ve had some ask me about it. who didn’t qualify, usually because they had a relative with lung cancer…. So, I really kind of rely on having it in my problem list and propagating forward as the easiest way to identify it.”*

(#17)

Providers felt that when patients understood the purpose of LCS and insurance coverage, they were more willing to agree to it. Providers in safety-net settings described deep patient trust as a motivator for screening but also noted that patients may rely heavily on provider recommendation without questioning or seeking additional information. This placed greater weight on the clarity of provider guidance and shared decision-making.

“For us at the FQHCS, our patients… have a lot of needs… And at the same time, their educational level is not as good sometimes…So, they really trust us and our recommendations. So, I try my best to not just impose my recommendations”

(#12)

Providers also observed a gap between LCS discussion and completion, with logistical challenges preventing some patients from following through. Competing priorities during visits (e.g., chronic disease management) limited time for preventive care conversations.

Providers also described their approach to LCS, including their knowledge of the process and logistics.

“I kind of make the assumption that [patients] haven’t had this conversation in the past. Most are pretty open to it. As far as me putting the orders in, I haven’t actually looked at how many actually complete it. But I would imagine, like most orders I put in, like them agreeing to do it and actually doing it, probably there’s a discrepancy.”

(#27)

“I think one of the main ways that I identify patients is by keeping track of their problem lists. When we have folks coming in, I do see a mostly older patient population… and so I’m managing a lot of different issues at a given time…. And, so those things get priority. Um, and therefore, things like cancer screening can fall to the background.”

(#17)

Providers emphasized that reminders, accurate EMR documentation, and streamlined referral processes may be essential for consistent screening uptake.

#### Provider Theme 2: Barriers at Every Level

Providers described barriers to LCS, often compounding one another. A key challenge was lack of infrastructure to support patients through screening. Providers noted the absence of dedicated navigators or centralized staff to assist with scheduling, insurance approvals, and follow-up, roles viewed as especially important for patients facing socioeconomic or linguistic barriers. Although some clinics had referral coordinators to verify insurance coverage, capacity was limited, and many patients lacked hands-on guidance to complete screening.

EMR-related challenges also contributed to missed opportunities. Although programmed to identify eligible patients, providers noted that EMR alerts were easy to overlook or inaccurate due to outdated or incomplete smoking histories. This reduced confidence in the EMR’s ability to support screening workflows and increased administrative burden as providers attempted to verify eligibility independently. One provider explains:

“The usual way that I do it is through the health maintenance alerts that we get in Epic. It will alert you because it already has the USPSTF guidelines embedded within the EMR. So that’s primarily what I use is to see who is due for a screening based on their smoking history and other things that qualify them. And then I have that conversation with them. But sometimes if they don’t have a smoking history in the chart, then it may not come up.”

(#44)

At the patient level, limited LCS awareness and competing priorities were common. Providers described patients who prioritized work, caregiving responsibilities, or urgent health conditions over preventive screening, especially in populations managing multiple comorbidities, limited English proficiency, or financial insecurity. Some patients expressed fears about radiation exposure or believed they were at lower risk if they had quit smoking, creating uncertainty about the need for LCS. The complexity of screening eligibility guidelines further added to the confusion, creating uncertainty for both patients and providers and leading to delayed or foregone screenings.

Insurance coverage was also described as a significant barrier. Providers noted inconsistent coverage across plans, delays in scheduling patients with public insurance, and lengthy wait times for screening appointments, which increased the likelihood of missed or forgotten visits.

“I guess insurance coverage is a major barrier. I don’t know the details of it, but apparently there is Cal-Optima that sometimes doesn’t cover LCS for patients, even though they definitely need it. ”

(#44)

Providers underscored the need for staffing solutions (e.g., navigation), improved EMR functionality to accurately identify eligible patients, and expanded patient education to address confusion and mistrust surrounding screening.

#### Provider Theme 3: I Appreciate the Help

Providers described Empower LCS as a valuable and timely tool for initiating LCS discussions, especially among patients who might otherwise be unaware or hesitant. A consistent benefit was that patients arrived to visits better informed, having reviewed the decision aid beforehand. Providers noted that this improved the quality of clinical conversations, reduced time spent explaining basic information and led to more patient-driven engagement around screening—a dynamic they rarely experienced in routine practice.

The intervention was seen as minimally disruptive to clinic workflows, and many providers highlighted the educational value it offered to patients new to the healthcare system. Because the decision aid and reminders were delivered outside of the clinic, they did not create significant operational burden. Several described the decision aid as clear and helpful in raising awareness and motivating screening conversations. Importantly, they believed the intervention helped normalize discussions of LCS, contributing to a more proactive culture around preventive care in family medicine settings.

“It is nice as the provider to have information relayed to me, while I can still take action at the time of the visit.”

(#27)

Providers identified opportunities to strengthen the intervention’s impact through better integration with clinical operations. Recommendations included using EMR messaging system instead of email, sending reminders closer to visits, and refining EMR alerts to improve the accuracy of eligibility notifications, which was limited by incomplete smoking histories.

Providers also expressed a desire for improved training and resources to support LCS counseling for themselves and their teams. Some emphasized the value of engaging family medicine residents more actively in the intervention and suggested applying similar approaches to other preventive screenings.

“We’re seeing patients every day, and sometimes things can fall through the cracks. And having a team that can make sure that patients are doing their screenings and letting them know they’re eligible for certain screenings can only be beneficial.”

(#14)

Several providers reflected the critical importance of building trust between patients and providers, particularly in underserved communities, and encouraged future efforts to include trust-building as a central focus. Overall, providers were supportive of continuing and scaling the intervention, with the caveat that improvements in communication, EMR functionality, patient engagement strategies, and provider education will be essential for sustained and broader impact.

## DISCUSSION

In this mixed-methods evaluation of a multilevel intervention to support LCS, both patients and PCPs viewed Empower LCS as acceptable, useful, and aligned with real-world clinical practice. Quantitative findings showed that most patients rated the intervention components helpful, and qualitative interviews revealed how the intervention increased awareness, motivation, and LCS-related conversations. Findings suggest that enhancing patient preparedness before visits, combined with provider-facing prompts, may help address persistent gaps in LCS uptake, particularly among underserved populations.

Our study showed patients’ screening experiences varied, with structural and emotional factors shaping patients’ overall impressions. Even when the procedure itself was quick and non-invasive, differences in setting, scheduling processes, and interpersonal interactions influenced patients’ confidence in the healthcare system and their willingness to follow up. Further, provider engagement was a key determinant of screening uptake, with patients viewing the clinical relationship, rather than patient self-advocacy, as the appropriate starting point for screening decisions.

Consistent with prior studies, patients described barriers to screening including difficulty navigating appointments and referrals, insurance authorization delays, transportation challenges, and smoking-related stigma (18,19). Some patients also reported low provider knowledge of LCS eligibility criteria and poor communication skills, echoing evidence linking low providers’ LCS guideline awareness to reduced LDCT ordering (4–8). Overall, these findings suggest that even motivated patients may face compounding barriers that slow or prevent LCS completion. Addressing a single barrier, such as patient awareness, may not be sufficient without concurrent efforts to simplify referral pathways, improve insurance-related navigation, and provide culturally and linguistically responsive support from the recommendation for screening through completion.

Provider interviews further contextualized these findings. PCPs reported that LCS conversations rarely originate from patients, making provider-led identification essential. However, competing demands during clinical encounters and inconsistent EMR data limited their ability to proactively identify eligible patients. Providers also highlighted common patient concerns including radiation exposure, work-related time constraints, and caregiving priorities that may reduce the salience of preventive screenings when immediate medical issues dominate the visit.

Patients and PCPs agreed that decision aids were a key component of Empower LCS in addressing barriers, increasing patient motivation, and saving provider time. Increasing patient awareness prior to appointments can facilitate shared decision-making during clinical encounters (20,21). Views on patient text reminders were mixed, but most supported provider reminders about eligibility, although some providers suggested these should be sent closer to the patients’ appointments. This study addes to existing knowledge by showing the feasibility and perceived value of Empower LCS, a multilevel intervention that engages both patients and primary care providers within routine clinical workflows.

Despite LDCT coverage since 2015, some patients were discouraged by authorization delays. Scheduling delays for LDCT and follow-up appointments with specialists were also common, consistent with previous studies (22,23). Some participants suggested implementation of mobile LCS units to increase access. Some patients also expressed dissatisfaction with follow-up after LDCT, particularly lack of clarity on results and next steps. Providers, therefore, suggested incorporating dedicated patient navigators to assist with scheduling, insurance issues, and other obstacles. These perspectives suggest that patient receptivity alone is insufficient without structured workflows to support identification, ordering, and completion of LCS.

### Limitations

This study has several limitations. The quantitative study sample size was relatively small as 40% (28/70) of Empower LCS participants did not complete the follow-up survey, raising concern for potential selection bias. Participants who completed follow up may differ systematically from non-responders in ways related to financial burden, health status, or engagement with care, which could influence the observed study outcomes. The reduced sample size also may have limited statistical power, limiting the generalizability of the results beyond the study population. In addition, only five primary care providers were interviewed due to the pilot nature of the study and resource constraints. While thematic saturation was reached for dominant provider perspectives, this small sample may not fully capture the range of perspectives across PCPs.

## CONCLUSION

Empower LCS was well received by patients and providers, and demonstrates a scalable approach to improving LCS awareness and motivation, shared decision-making during primary care visits, and supporting guideline-based screening. The intervention integrates into existing primary care workflows through automated EMR prompts and patient-facing decision aids without requiring substantial new resources. Practical implementation should prioritize standardized EMR smoking documentation during rooming or patient portal check-in to improve eligibility identification. Incorporating navigation support can further address structural, linguistic, and financial barriers, particularly for underserved populations. Targeted outreach strategies will be essential to ensure equitable screening uptake.

## Supplementary Material

supplementary Materials

## Figures and Tables

**TABLE 1.** Characteristics of Patient Participants

Characteristics	Participants in Quantitative Analysis (N = 41)	Participants in Qualitative Analysis (N=11)
Mean Age ± SD [Range]	61.7 ± 6.4 [51,77]	59.4 ± 4.5 [51,65]
Gender		
Female	15/41 (36.6%)	3/11 (27.3%)
Male	26/41 (63.4%)	8/11 (72.7%)
Race		
Asian	5/41 (12.2%)	4/11 (36.4%)
Black	1/41 (2.4%)	0/11 (0.0%)
Other^a^	16/41 (39.0%)	1/11 (9.1%)
White	19/41 (46.3%)	6/11 (54.5%)
Ethnicity		
Hispanic or Latino	19/41 (46.3%)	5/11 (45.5%)
Not Hispanic or Latino	22/41 (53.7%)	6/11 (54.5%)
Primary Language		
English	18/41 (43.9%)	5/11 (45.5%)
Spanish	18/41 (43.9%)	5/11 (45.5%)
Vietnamese	5/41 (12.2%)	1/11 (9.1%)
Marital status		
Married or living with a partner	20/41 (48.8%)	6/11 (54.5%)
Single, never married, divorced, separated, widowed	21/41 (51.2%)	5/11 (45.5%)
Employment		
Full time, part time, self-employed	15/41 (36.6%)	6/11 (54.5%)
Not employed	26/41 (63.4%)	5/11 (45.5%)
Health insurance		
Medicare and military	7/41 (17.1%)	3/11 (27.3%)
Medicaid	25/41 (61.0%)	3/11 (27.3%)
Private	6/41 (14.6%)	5/11 (45.5%)
Uninsured or self-pay	3/41 (7.3%)	0/11 (0.0%)
Education		
Did not graduate high school	12/38 (31.6%)	3/11 (27.3%)
High school graduate	7/38 (18.4%)	4/11 (36.4%)
Some college or technical school	9/38 (23.7%)	1/11 (9.1%)
Graduated from college or technical school	10/38 (26.3%)	3/11 (27.3%)
Self-reported smoking status		
Current	15/41 (36.6%)	3/11 (27.3%)
Former	26/41 (63.4%)	8/11 (72.7%)

a American Indian, Alaska Native, Native Hawaiian, Pacific Islander, Other, or Prefer Not to Say

**TABLE 2.** Patient Interviews Parent Codes

Parent Code	Description
*Patient Views on Current LCS Status*	Most viewed LCS as important and were eager to discuss it with their providers. Motivations included long-term smoking history, family history of lung cancer, occupational exposures, and a desire for early detection and peace of mind. Among those with positive LCS results, some reported barriers and frustrations, including difficulty finding a lung surgeon who accepted Medi-Cal, leading to prolonged, unnecessary hospitalization.
*Patients’ Perceptions of Providers*	Some expressed satisfaction with their providers’ communication, attentiveness, and a proactive approach to LCS. Others felt providers were hesitant to fully engage in LCS discussions, due to insurance issues and time constraints during visits.
*Multi-level Barriers to LCS*	Patient-reported barriers to LCS included system issues such as insurance authorization delays, perceived stigma, and language barriers.
*Patients’ Reflections on the Empower LCS Intervention*	Study information increased participants’ awareness, motivated them to pursue LCS, and encouraged them to educate friends and community members. Some viewed patient text reminders as an effective and accessible method of communication particularly in underserved communities; while others found them less impactful, as they were already proactive in scheduling their screenings. Participants broadly supported the idea of healthcare providers receiving reminders to discuss LCS with patients.
*Participants’ Suggestions*	Organizing mobile LCS units to facilitate easier access for screenings without extensive delays. Improving provider competence and communication skills about LCS eligibility. More information about longer term screening recommendations and interpreting consistent negative results over time.

**TABLE 3.** Primary Care Provider Interviews Parent Codes

Parent Code	Description
*Provider Views on Current LCS Status*	Discussion of smoking history during patient encounters to determine LCS eligibility occurs routinely by most providers. Although some providers mentioned EMR supposedly flagged eligible patients, a few of them could not recall any specific instances of actually noticing them. Some had concerns about inaccuracies in eligibility flags within the EMR due to inaccurate structured smoking history. Providers generally found patients receptive to LCS and believed most would proceed with scheduling when covered by insurance.
*Multi-level barriers to LCS*	Patients’ lack of awareness about LCS, insurance issues, patients not prioritizing LCS over work and other commitments, and having strong concerns regarding radiation exposure. Though most providers found the scheduling process straightforward, some identified the lack of dedicated patient navigators to help regarding both scheduling and insurance issues as a major shortcoming.
*Providers’ Reflections on the Empower LCS Intervention*	Intervention was viewed as valuable and timely with a strong potential to increase LCS rates by increasing patients’ awareness. Intervention did not disrupt the practice’s workflow and was even time savings by providing pre-appointment information to patients. Intervention was suitable for family medicine practices and should continue, especially with efforts to enhance patient education and improve EMR alert accuracy.
*Participants/ Suggestions*	Sending reminders closer to the patients’ appointments Incorporating LCS as a measure within clinic evaluation systems Refining Epic health maintenance flags Increasing outreach to family medicine residents who are further along in their training Enhancing the patient’s ability to schedule screenings online.

## Data Availability

Individual de-identified participant data (including data dictionary) can be accessed via direct request to corresponding author and establishing a data use agreement.

## APPENDIX A. SUPPORTING INFORMATION

Supplemental data associated with this article can be found in the online version at doi:10.1016/j.acra.2026.03.014.

## Abbreviations:

EMR: Electronic medical record
FQHC: Federally qualified health center
HIPAA: Health Insurance Portability and Accountability Act
HRSNs: Health-related social needs
LDCT: Low-dose CT
LCS: Lung Cancer Screening
PCP: Primary Care Provider
RE-AIM: Reach, Effectiveness, Adoption, Implementation and Maintenance
USPSTF: U.S. Preventive Services Task Force
