# Supplementary material for: Patients’ and Providers’ Perspective of a Multi-level Approach to Improve Participation in Low-dose CT for Lung Cancer Screening (Empower LCS): A Mixed-Methods Analysis
Source: Acad Radiol. Author manuscript; Available in PMC 2026 Apr 15. (PMC13077605; doi:10.1016/j.acra.2026.03.014)
Supplement: supplementary Materials [file NIHMS2163863-supplement-supplementary_Materials.docx]

**Patients’ and Providers’ Perspective of a Multi-level Approach to Improve Participation in Low-dose CT for Lung Cancer Screening (Empower LCS): A Mixed-Methods Analysis**

**Supplementary Material 1. Empower LCS – Patient Interview**

**Supplementary Material 2. Empower LCS- Provider Interviews**

**Supplementary Material 1. Empower LCS – Patient Interview**

**Introduction**

Thank you for participating in this discussion. My name is [Interviewer Name]. I am one of the research team members for the Empower LCS study that you are enrolled in. We want to learn more about your perceptions about lung cancer screening as well as your experience with the study intervention. Specifically, we want to know what worked well and what could be improved.

**General Questions about getting lung cancer screening**

To get us started, please tell me a bit about you.

First, I would like to start by asking you some general questions about your experience with lung cancer screening.

[FOR PATIENTS WHO HAD DISCUSSION ABOUT LCS WITH THEIR PROVIDER BASED ON SURVEY]

On your recent survey, you indicated that you talked about lung cancer screening with your healthcare provider. Tell me about that experience.

- How did it go?
- Who initiated the conversation about lung cancer screening?
- In what ways would you like to have had that go differently? (if anything)

[FOR PATIENTS WHO COMPLETED THE LCS BASED ON SURVEY]

You also indicated that you completed a lung cancer screening exam. Tell me more about what made you make a decision to get the screening.

- How was your experience getting tested?
- What was the result of your lung cancer screening CT scan?
- Did your doctor reached out to you to explain your lung cancer screening CT results? How did that conversation go?
- Tell me, what was explained to you as the next steps.

[FOR PATIENTS WHO HAVE SCHEDULED THE LCS BUT NOT COMPLETED YET]

You indicated that you have scheduled your lung cancer screening but not completed it yet. Tell me more about what led you make the decision to schedule the test.

- What did expect or want from the screening process?

[FOR PATIENTS WHO HAVE NOT SCHEDULE THE LCS]

You also indicated that you have not scheduled your lung cancer screening yet. Tell me more about why not?

- What has influenced your decision? Where did you get information?
- What concerns or worries do you have about screening? Are you planning to complete your lung cancer screening CT in future?

[FOR PATIENTS WHO DID NOT HAVE A DISCUSSION ABOUT LCS WITH THEIR PROVIDER BASED ON SURVEY]

On your recent survey, you indicated that you did not talk about lung cancer screening with your doctor. Tell me more about why not.

- What were the barriers? PROBE: not having an encounter with the physician, encounter being too short, healthcare provider feeling rushed, patient not being comfortable bringing up the discussion, having concerns about lung cancer screening etc
- What could have been different for you to have the discussion?
- Are you planning to complete your lung cancer screening CT in future? If not, why not?

**Experience with the Empower LCS intervention**

Now, I would like to ask you some questions regarding the information you received as part of the study. If you remember, we sent you an information booklet about benefits, and risks of lung cancer screening and its insurance coverage. Also some people received information booklet about the Patient Advocate Foundation, a national financial navigation program. We also reminded both you and your primary care provider to talk about lung cancer screening during your visit. When I say study intervention, I mean all of these steps you received.

1. What was your overall experience with the study intervention?
   1. Did you feel that the intervention was able to address your concerns or needs?
      - If yes, how so?
      - If no, why not?
2. Which part of the intervention did you find most helpful? Information booklet on benefits, risks, and insurance coverage of lung cancer screening, information about patient advocate foundation if you received one, the reminder on talking about lung cancer screening with your provider.
   1. Why did you [insert participant answer] find that most helpful?
3. How was your experience with the information booklet about lung cancer screening?
4. How did it feel receiving a text message to reminder you to discuss getting a lung cancer screening with your primary care provider prior to your scheduled appointment?
   1. Did you find the text message helpful? If yes, how was it helpful? If no, why not?
   2. What do you think of the timing of the text message in relation to your appointment?
5. How do you feel about the fact that your primary care provider also received a reminder to talk about lung cancer screening with you? Do you see any value in it? If yes, why? If not, why not?
6. If you were to come up with a program to help patients get their annual lung cancer screening what would you do?
7. Is there anything else you would add to our conversation today about getting lung cancer screening when you are due for one?

Thank you so much for your time and participation.

**Supplementary Material 2. Empower LCS- Provider Interviews**

**Introduction**

Thank you for participating in this discussion. My name is [Interviewer Name]. We want to learn more about your experience with the Empower LCS intervention, which includes notifying primary care providers of their patients eligibility for lung cancer screening within 2 weeks prior to the patients’ appointment. This intervention was recently completed for some of your patients who were enrolled in the Empower LCS study. Specifically, we want to know what worked well and what could be improved for patients and providers participating in this intervention.

**Overview of Provider Role and Usual Care**

First, I would like to ask you some general questions about your role and standard care at your practice.

1. What is your current role? (MD, NP, resident, fellow)
2. How many years of experience do you have in your current position?
   1. Years of experience at the current practice?
3. What is the usual care at your practice for identifying patients eligible for lung cancer screening and discussing with them?
4. Do you or anyone from your team updates patients’ accurate smoking history in EMR or make it easy to identify those eligible for lung cancer screening?
5. Per institutional IT, currently patients who are eligible for lung cancer screening are flagged in the EMR. Have you ever noticed this flag for your patients?
   1. If yes: do you find them useful?
   2. If yes: How do you follow up on them?
6. What are some barriers that your patients may have to get lung cancer screening?
7. Does your practice has a navigator to help patients schedule their lung cancer screening if you order one?

**Experience and Satisfaction with the Empower LCS Intervention**

Next, I would like to ask you some questions regarding your experience with the Empower LCS intervention and what went well and what could be improved.

*Overview*

1. Can you describe your perception of the purpose or goal of the Empower LCS intervention?
   1. Do you recall the different components of the intervention and their purpose? (if they don’t know, we can briefly mention, it involves giving patients an information sheet about risks, benefits and insurance coverage for lung cancer screening, an information sheet on a national financial navigation program if patient screens positive for a health-related social risk, and reminder of both patients and providers of patients eligibility for lung cancer screening within 2 weeks prior to their PCP appointment)

*Reach*

1. Do you think the Empower LCS intervention is applicable and timely for your patients?
   1. If yes, how so?
   2. If not, why not?

*Effectiveness*

1. To what extent do you think patients benefitted from Empower LCS intervention?
   1. Probe: Better adherence to lung cancer screening?
   2. If indicate patients did not benefit: Why do you think the intervention was not beneficial?
2. Did the patients who participated in the intervention started any conversation about getting lung cancer screening CT during their visit with you?
   1. If yes, did patient had any concerns or barriers?
   2. If yes, did you have any concerns or barriers?
3. If patient did not start any conversation, did you started conversation about getting lung cancer screening CT?
   1. If yes, did patient had any concerns or barriers?
   2. If yes, did you have any concerns or barriers?

*Adoption*

1. Do you think the Empower LCS intervention is suitable for your practice?
   1. Why or why not?

*Implementation*

1. How did the steps in Empower LCS intervention that you were involved with affect your practice workflow?
   1. How long did it take to have discussion about lung cancer screening with each patient?
2. What were some factors that facilitated intervention delivery?
3. What barriers did you face in delivering the intervention?
4. Do you think the Empower LCS intervention complicates the primary care visit?
   1. If yes, how so?

*Maintenance*

1. Would you like for the system to continue offering the Empower LCS intervention?
   1. If yes, what factors influenced that decision?
   2. What would help you maintain the intervention (i.e., resources needed for sustainability)?
   3. If not, please explain:
      1. What changes would you make so that the practice may continue offering the Empower LCS intervention (e.g., staffing, funds, workflow changes, etc.)?
2. Is there anything else that you would like to add about the delivery of the Empower intervention?

Those are all the questions I have. Thank you so much for your time.
